# Can a Novel Web-Based Computer Test Predict Poor Simulated Driving Performance? A Pilot Study With Healthy and Cognitive-Impaired Participants

**DOI:** 10.2196/jmir.2943

**Published:** 2013-10-18

**Authors:** Tobias Nef, René M Müri, Rahel Bieri, Michael Jäger, Nora Bethencourt, Ioannis Tarnanas, Urs P Mosimann

**Affiliations:** ^1^Gerontechnology and Rehabilitation GroupUniversity of BernBernSwitzerland; ^2^ARTORG Center for Biomedical Engineering ResearchUniversity of BernBernSwitzerland; ^3^Division of Cognitive and Restorative NeurologyDepartment of NeurologyUniversity Hospital Inselspital, University of BernBernSwitzerland; ^4^Department of Old Age PsychiatryUniversity Hospital of PsychiatryUniversity of BernBernSwitzerland

**Keywords:** cognitive impairment, Web-based cognitive test, computer-based tests, driving simulation

## Abstract

**Background:**

Driving a car is a complex instrumental activity of daily living and driving performance is very sensitive to cognitive impairment. The assessment of driving-relevant cognition in older drivers is challenging and requires reliable and valid tests with good sensitivity and specificity to predict safe driving. Driving simulators can be used to test fitness to drive. Several studies have found strong correlation between driving simulator performance and on-the-road driving. However, access to driving simulators is restricted to specialists and simulators are too expensive, large, and complex to allow easy access to older drivers or physicians advising them. An easily accessible, Web-based, cognitive screening test could offer a solution to this problem. The World Wide Web allows easy dissemination of the test software and implementation of the scoring algorithm on a central server, allowing generation of a dynamically growing database with normative values and ensures that all users have access to the same up-to-date normative values.

**Objective:**

In this pilot study, we present the novel Web-based Bern Cognitive Screening Test (wBCST) and investigate whether it can predict poor simulated driving performance in healthy and cognitive-impaired participants.

**Methods:**

The wBCST performance and simulated driving performance have been analyzed in 26 healthy younger and 44 healthy older participants as well as in 10 older participants with cognitive impairment. Correlations between the two tests were calculated. Also, simulated driving performance was used to group the participants into good performers (n=70) and poor performers (n=10). A receiver-operating characteristic analysis was calculated to determine sensitivity and specificity of the wBCST in predicting simulated driving performance.

**Results:**

The mean wBCST score of the participants with poor simulated driving performance was reduced by 52%, compared to participants with good simulated driving performance (*P*<.001). The area under the receiver-operating characteristic curve was 0.80 with a 95% confidence interval 0.68-0.92.

**Conclusions:**

When selecting a 75% test score as the cutoff, the novel test has 83% sensitivity, 70% specificity, and 81% efficiency, which are good values for a screening test. Overall, in this pilot study, the novel Web-based computer test appears to be a promising tool for supporting clinicians in fitness-to-drive assessments of older drivers. The Web-based distribution and scoring on a central computer will facilitate further evaluation of the novel test setup. We expect that in the near future, Web-based computer tests will become a valid and reliable tool for clinicians, for example, when assessing fitness to drive in older drivers.

##  Introduction

### Cognition and Driving

Driving a car is a very challenging instrumental activity of daily living that requires the integration of high-level cognition, vision, and motor function [1]. These three domains are usually evaluated when assessing medical fitness to drive in older drivers, as they are commonly affected by age-related diseases [2]. In this article, we focus on the assessment of driving-relevant cognition in older drivers.

Since driving is a complex activity, driving performance is very sensitive to cognitive impairment [3], which is commonly the result of age-related neurodegenerative disorders (eg, Alzheimer’s disease and other causes of dementia) [4]. The prevalence of neurodegenerative disorders doubles every five years after the age of 65 years [5]. Therefore, health professionals need easy access to screening tests in order to assess fitness to drive. Due to the ageing population in the Western world and increasing numbers of older drivers, identifying drivers at risk without unnecessarily restricting others is a challenging but important task [6]. This task requires reliable and valid cognitive screening tests with good sensitivity and specificity to identify at-risk drivers.

### Testing Driving Performance

On-the-road testing (ORT) has been suggested as being a reasonable proxy measure for naturalistic driving in older adults with a range of cognitive impairments [7]. It is the gold standard for measuring driving performance and several authors suggest using it to assess fitness to drive in older drivers [7-10]. Despite its advantages, ORT has limitations: it is time consuming [11] and may have adverse effects that could lead to dangerous driving situations [12]. In addition, researchers cannot control for environmental conditions such as light, weather, traffic, and pedestrians [13].

That is why more recently, driving simulators (DS) have been recommended as a proxy measure for naturalistic driving and they have been introduced to assess fitness to drive of older drivers with and without cognitive impairment [14,15]. Simulators have the advantage of being intrinsically safe, providing excellent controllability, reproducibility, and standardization. Furthermore, they can be installed in specialist centers, their use is less time consuming, and they require fewer organizational demands than ORT. Several studies demonstrate the validity of DS as a proxy for naturalistic driving [13,16-19]. Disadvantages of DS are that they are expensive and large, both of which reduce their accessibility to primary care physicians and older drivers. Furthermore, there is a lack of standard test protocols and cutoff values. Finally, simulator sickness is a rather common side effect, especially for older female drivers. This interferes with DS driving performance [20].

### Web-Based Computer Testing of Driving Performance

In a recent study, Rockwood et al [21] used a Web-based dataset to determine the level of cognitive impairment of older Internet users. They concluded that online tracking of people with cognitive impairment can be used to stage dementia. For review, see [22]. Also, when measuring driving performance, some of the limitations of DS can be resolved by the introduction of computer tests. By using standard personal computers with cheap off-the-shelf interface components similar to those that are used in computer gaming, cheap and easily accessible computer tests can be implemented [23]. Compared to DS, they can be easily integrated into the physician’s office. Web-based computer tests use the World Wide Web to distribute the software that can run on local client computers in the physician’s office. Moreover, with Web-based computer tests, the scoring of user performance can be conducted on a central server computer. This allows for generation of a dynamically growing database with normative values and ensures that all users have the same up-to-date normative values. This concept has been successfully introduced by Mills et al [24] for the application of a Web-based computer test to assess driving performance under alcohol and drug influence. The authors mention central scoring algorithms and a central database with normative values as the main advantage of Web-based computer tests. That is why we hypothesized that Web-based computer tests would also be helpful to assess fitness to drive of older drivers and we have developed the Web-based Bern Cognitive Screening Test (wBCST) to assess driving-relevant cognitive performance. The novel wBCST is based on a previously developed computer test [25].

In this pilot study, we investigate whether or not the novel wBCST correlates with DS performance and whether it is able to differentiate between participants with poor and good simulated driving performance. To have a broad and diverse test population, we recruited younger and older healthy participants as well as older participants with cognitive impairment for this study. Hence, this paper first describes the novel wBCST and the DS used, followed by a correlation analysis and a receiver operating characteristics (ROC) [26] analysis to calculate sensitivity and specificity of the novel test to predict driving simulator performance. The discussion and conclusion outline advantages and disadvantages of the wBCST and present possible future applications and research directions.

##  Methods

### Participants

Thirty healthy younger adults (age 22-40 years), 60 healthy older adults (age >50 years), and 15 older (age >50 years) participants with cognitive impairment (Montreal Cognitive Assessment Score [MoCA] <26) [27] were recruited by advertisements in local newspapers and within the local memory clinic. All participants were required to have had a driver’s license for at least two years and to have been driving during the last two years. Exclusion criteria for the study were visual impairment (corrected far visual acuity <0.5 degrees, near visual acuity <0.8 degrees) or significant motor impairment (timed-up-and-go-test >12 seconds) [28]. The study was carried out in accordance with the Declaration of Helsinki and was approved by the local ethics board. Written informed consent was obtained from all participants prior to inclusion. No compensation for participation was provided. Six participants were excluded due to visual impairment. Eighteen participants stopped the DS drive due to simulator sickness (four younger, nine older, and five participants with cognitive impairment). Their data were excluded from further analysis. Due to a malfunctioning of the DS, the data of one healthy older test person were not recorded. The data of the remaining 80 participants were included in the analysis. There were 26 young (range 22-39 years, mean 29.4 years, SD 4.7 years), 44 healthy older (range 54-85 years, mean 68.4 years, SD 5.5 years), and 10 impaired older participants (range 55-87 years, mean 72 years, SD 9.6 years). Trail Making Test A (TMT-A) [29], Trail Making Test B (TMT-B) [29], the MoCA score [27], clock-drawing test (CDT) [30], and timed-up-and-go test [28] were assessed to characterize the participants.

### Web-Based Computer Test

**Figure 1:**
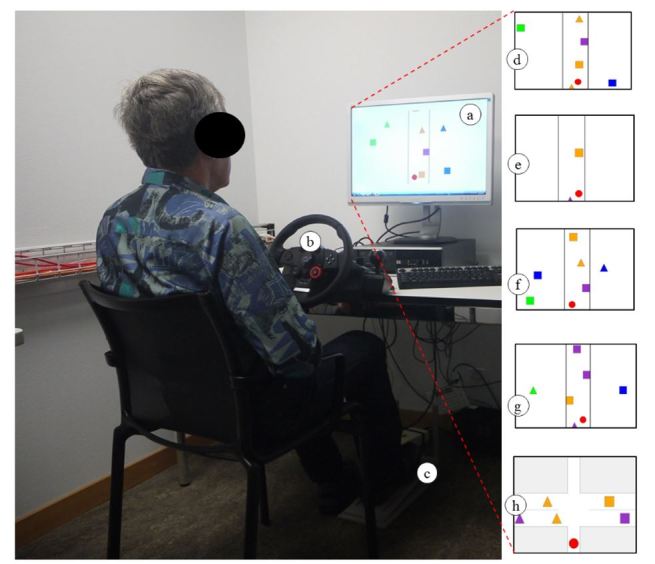
Older participant during the wBCST evaluation. A 24-inch monitor (a) is used to present the test material and participants interact with the system via a steering wheel (b) and foot pedal (c). Each subtest uses similar graphical objects as shown in the screenshots (d-h).

A literature review of the most important driving-relevant cognitive functions and how they are affected by cognitive impairment was conducted [6]. This analysis was used to develop a novel computer test [25], which we extended in the context of this study to the wBCST. The wBCST measures eye-hand coordination, selective attention, divided attention, executive function, distance judgment, and speed regulation. It is composed of five subtests, each measuring one of the before-mentioned cognitive competencies. The setup of the wBCST comprises a computer screen showing the test scenario (240B1CS/00 24 inc, Philips Inc), a steering wheel (Driving Force GT, Logitec Inc) with foot pedal, and a personal computer with Windows 7 (Microsoft Inc) operating system. Figure 1 shows a healthy test person taking the wBCST and screenshots of the visual representation of the five subtests. The test persons interact with the wBCST via steering wheel and foot pedal.

Subtest 1 measures selective attention with a visual scene consisting of a simplified street in the center of the screen (Figure 1d) with objects moving from the top down. A red dot moves automatically in the horizontal direction to avoid collisions with oncoming objects and with the roadside. The user is instructed to not touch the steering wheel, but to press the foot pedal whenever a visual target (blue square) appears in the periphery (Figure 1d). In subtest 2, eye-hand coordination is measured and the same street is presented. The test person must use the steering wheel to control the horizontal position of the red dot to avoid collisions with the other objects and with the roadside (Figure 1e). There is no peripheral subtask in this test. Subtest 3 measures divided attention and both tasks of subtests 1 and 2 need to be carried out simultaneously; hence, the user must steer (central task) and react to peripheral stimuli (peripheral task) (Figure 1f). Subtest 4 is designed to measure executive functions and the user must react to more complex peripheral stimuli (green triangle and blue square) while ignoring the movement in the center of the screen (Figure 1g). In subtest 5, distance judgment and speed regulation are assessed. The user can control the velocity of a red dot with the foot pedal. The task as shown in Figure 1h is to cross intersections without colliding with the horizontally moving objects. During the tests, the false positive and false negative errors of the peripheral target detection task and the number of collisions with moving objects as well as with the street border are recorded. The test duration is about three minutes per subtest. With instruction, administration of the entire wBCST takes roughly 20 minutes. For videos of the subtests, see Multimedia Appendices 1 and 2.

### Drive in the Virtual Reality Driving Simulator

**Figure 2:**
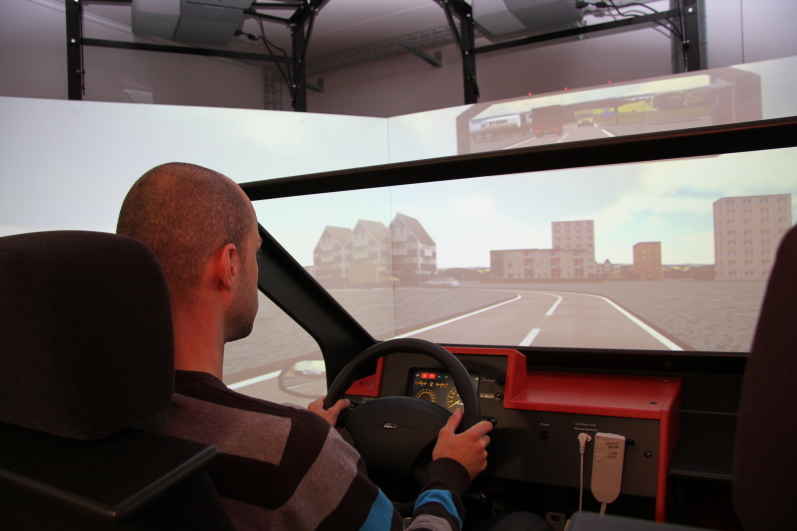
High-fidelity fixed-frame driving simulator with younger test subject. The steering wheel, cockpit, and parts of two projection screens with the virtual driving screen are shown.

A commercially available high-fidelity fixed-frame driving simulator (F12PI-3/A88, Foerst GmbH) with a custom-built virtual driving circuit was used to measure DS performance. A virtual scene was projected by three projectors (Ultra-Short focus LCD projectors, Sanyo) with 1024x786 pixel resolution onto three projection screens (1.8 x 1.4 m) that were installed in front of the driver. DS components utilized by the participants were steering wheel, brake and gas pedals, rear and side mirrors, and turn signals (Figure 2). The virtual driving scene consisted of a street with two lanes in each direction in a suburban environment. The test drive included two left turns at intersections: one with traffic crossing left to right and one with oncoming traffic. Furthermore, it comprised a construction area with road work on both lanes, a roundabout with an unexpected cyclist, a deer hiding behind trees and suddenly jumping onto the road, a car unexpectedly leaving its parking lot, and a child running into the street after a ball. The instruction was to respect traffic rules and to drive as if in a real-world environment.

Participants performed a familiarization run (three minutes) to get used to the handling of the simulator and a test drive (six minutes) during which data were recorded. Once the end of the track was reached, the car stopped automatically. If a participant felt uncomfortable, the DS was stopped. Primary outcome measure of the driving simulator was the number of errors, *E*_*DS*_ (ie, collisions, traffic rule violations, driving in the wrong traffic lane). Secondary outcome measures were mean speed variability, mean lateral acceleration, cumulated time spent on brake, distance to collision, and time to collision. Distance and time to collision are defined by how long (time, respective distance) the virtual car could continue on its current path with constant velocity until a collision would occur.

### Statistical Analysis

The results of the wBCST were ranked and scored. Thus, the score, *S*_*i*_, of each subtest was calculated as *S*_*i*_ *= (N−rank(E*_*i*_ *)) / (N−1)* with *N* being the total number of test samples and *E*_*i*_ being the number of errors in the subtest. With this formula, 1 corresponds to the best possible test results and 0 the worst. Subtest 3 reveals two results, *S*_*3p*_ for the peripheral task and *S*_*3c*_ for the central task. The overall test result, *S*_*wBCST*_, was calculated as the mean value of S_*1*_ to *S*_*5*_. The same formula was also used to score the performance in the DS—namely, the score, *S*_*DS*_, that was calculated out of *E*_*DS*_, as well as the scores for speed variability, lateral acceleration, time on brake, distance to collision, and time to collision.

Pearson product-moment correlations, step-wise regressions, and associated tests of significance were calculated across *S*_*DS*_, *S*_*wBCST*_, and the other secondary measures. The statistical significance of the correlation was computed by transforming the correlation matrix to create a *t* statistic having n-2 degrees of freedom where n was the number of observations.

In a second step, the number of errors *E*_*DS*_ in the driving simulator was used as a classification criterion to divide the participants into two groups. As suggested by others [31], the mean value plus 1 SD was used as cutoff for one group with good DS performance and another group with poor DS performance. The mean values of the DS and the wBCST for both groups were calculated and the significance of the differences was calculated using a non-parametric Mann-Whitney U-test. Hypotheses were one-sided. Furthermore, a ROC analysis was conducted to determine the sensitivity and specificity of the wBCST when predicting DS performance. A Matlab (The MathWorks Inc) algorithm based on the work of Hanley et al [32] and implemented by Cardillo et al [33] was used to calculate the ROC curve and the area under the ROC curve (AUC). The Wilcoxon test was used to calculate whether the difference from a random classifier was significant. Also, the sensitivity, specificity, and efficiency (fraction of subjects that are correctly classified) were computed for different cutoff values.

## Results

### User Statistics

The data of 80 participants were included in the data analysis. Ten were assigned, based on the number of errors in the DS using mean value + 1 SD cutoff, to the poor DS performance group (*E*_*DS*_≥4) and 70 were assigned to the good DS performance group (*E*_*DS*_<4). With this criterion, all younger participants, 86% (38/44) of the healthy older, and 60% (6/10) of the older participants with cognitive impairment were classified into the good DS group. The mean test performance in MoCA, TMT-A, TMT-B, CDT, and timed-up-and-go-test of the participants with good DS results was better compared to the other group. These differences were statistically significant.

**Table 1:** User statistics of all participants and the two subgroups.

	All Participants, n=80	Participants with good DS^a^ performance, n=70	Participants with poor DS performance, n=10	Significance of group difference
Young (<40 years), n	26	26	0	
Older (>50 years), without cognitive impairment (MoCA^b^≥26), n	44	38	6	
Older (>50 years), with cognitive impairment (MoCA<26), n	10	6	4	
MoCA, score (SD)	28.2 (2.9)	28.5 (2.8)	26.2 (3.1)	*U*_70,10_=179.00, *P*=.004
TMT^c^ A, seconds (SD)	28.0 (14.4)	26.9 (14.2)	35.7 (14.1)	*U*_68,9_=535.00, *P*<.001
TMT B, seconds (SD) [30]	71.6 (52.5)	68.8 (53.2)	91.2 (45.7)	*U*_68,9_=479.00, *P*=.003
CDT, score (SD)	6.2 (1.9)	6.5 (1.6)	4.3 (3.1)	*U*_70,10_=170.00, *P*<.001
Timed-up-and-go-test, seconds (SD)	6.9 (3.2)	6.8 (2.9)	7.5 (4.7)	*U*_63,8_=420.50, *P*=.001

^a^DS: driving simulator

^b^MoCA: Montreal Cognitive Assessment

^c^TMT: trail making test (A and B)

^d^CDT: clock drawing test

### Correlation Analysis of wBCST and DS Performance

**Figure 3:**
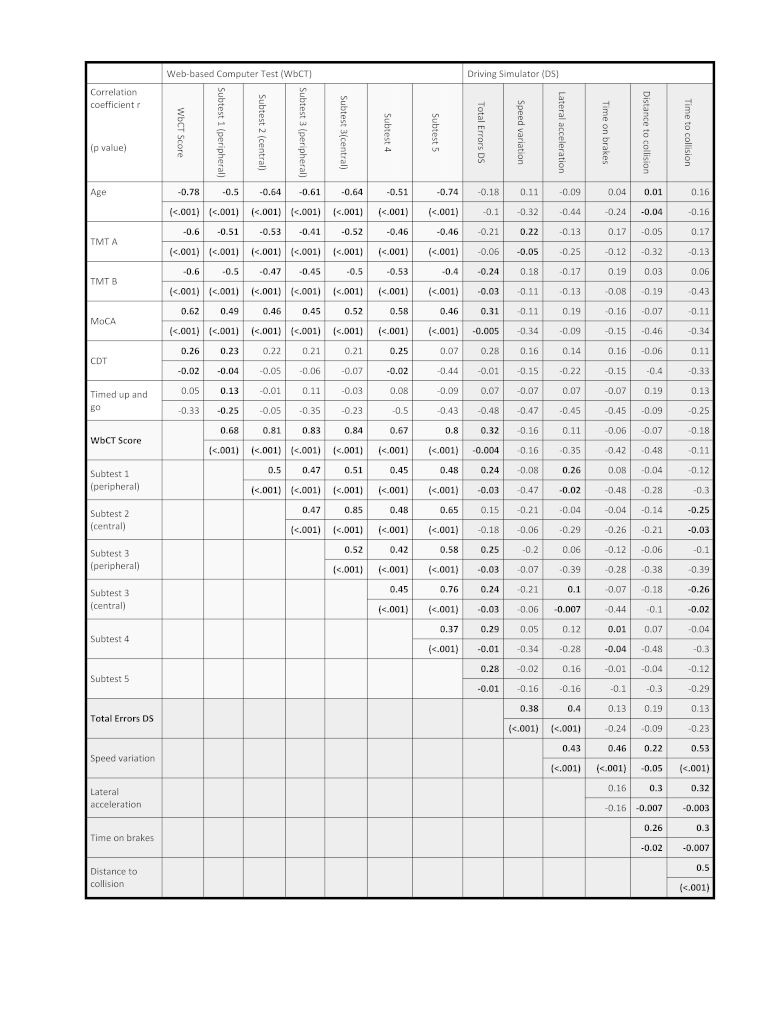
Correlation analysis of the wBCST and the DS. The table shows the Pearson product-moment correlation coefficient *r* and the associated *P* values in parentheses. Correlations with *P*<.05 are indicated in bold font.

Figure 3 shows the results of the correlation analysis with the correlation coefficient *r* and the associated *P* values. The wBCST score, *S*_*wBCST,*_ correlates with the DS score, *S*_*DS,*_ with *r*=0.32 (*P*=.004). The individual results of the wBCST subtest, *S*_*1…6*_, correlate with the overall score, *S*_*wBCST*_, with *r* values varying between 0.68-0.83 with *P*<.001. In the DS, the overall score *S*_*DS*_ correlates with the speed variation with *r*=0.38 (*P*<.001), the lateral acceleration with *r*=0.40 (*P*<.001), the time spent on the brake pedal *r*=0.13 (not significant, *P*=.24), the distance to collision with *r*=0.19 (not significant, *P*=.19), and the time to collision *r*=0.31 (not significant, *P*=.23).

### Sensitivity and Specificity of the wBCST in Predicting DS Performance

**Figure 4:**
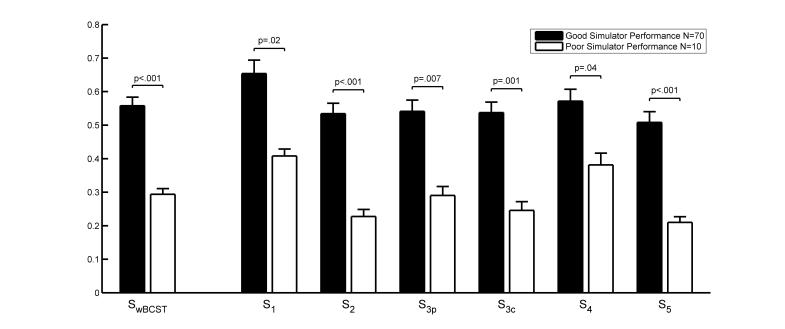
Web-based Bern Cognitive Screening Test (wBCST) performance of the group with good simulated driving performance (n=70) and group with poor simulator performance (n=10). All values are normalized and ranked. The score S_wBCST is the mean value of the subscores S (1…5). Subtest 3 is represented with two entries, S_3p for the peripheral recognition task and S_3c for the central steering task. Bars indicate the standard error.

The ranked normalized wBCST performances of the two groups are represented in Figure 4. The group with poor DS performance performed less well in all tests. The group differences are significant for all subscores and highly significant (*P*<.001) for the overall *S*_*wBCST*_ score, subtest 2 (*S*_*2*_), and subtest 5 (*S*_*5*_).

**Figure 5:**
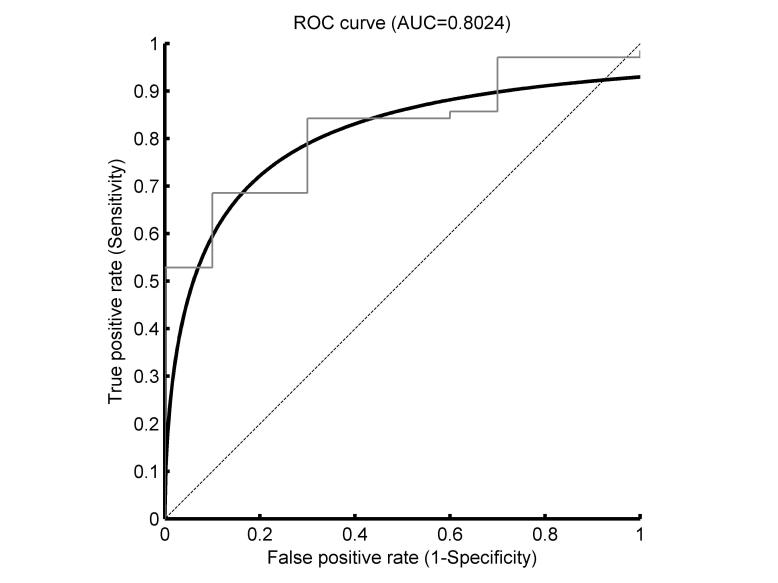
Receiver operating characteristics (ROC) curve for using S_wBCST to predict SD driving performance, respectively to differentiate between subjects with good and poor DS performance. The thin gray line is the empirical curve, the solid black line is the smoothed (Gaussian-based) curve, and the dotted diagonal line indicates no discrimination (50% chance).

The ROC curve for using *S*_*wBCST*_ to predict DS performance is shown in Figure 5. The AUC=0.80 is significantly better (*P*<.001) than a random classifier. The 95% confidence interval of the AUC is 0.68-0.92. A selection of possible cutoff scores and the corresponding sensitivity, specificity, and efficiency values is presented in Table 2.

**Table 2:** Receiver operating characteristic (ROC) curve analysis: possible cutoff values and corresponding sensitivity, specificity, and efficiency.

Cutoff	Sensitivity	Specificity	Efficiency
0.88	0.94	0.30	0.86
0.84	0.91	0.30	0.84
0.83	0.89	0.30	0.81
0.81	0.86	0.30	0.79
0.77	0.84	0.50	0.80
0.75	0.83	0.70	0.81
0.73	0.80	0.70	0.79
0.68	0.77	0.70	0.76
0.66	0.74	0.70	0.74
0.65	0.71	0.70	0.71
0.64	0.69	0.70	0.69
0.63	0.69	0.90	0.71
0.61	0.66	0.90	0.69
0.57	0.63	0.90	0.66
0.56	0.60	0.90	0.64
0.53	0.57	0.90	0.61
0.52	0.54	0.90	0.59
0.51	0.53	0.90	0.58

##  Discussion

### Principal Results

When using the criteria proposed by Cohen [34], the correlation between *S*_*DS*_ and the total score *S*_*wBCST*_ has a medium effect size (*r*>0.3) and is significant with *P*=.004, which supports the hypothesis that the wBCST correlates with DS performance. Very good correlations with large effect sizes (*r*>0.5) were found among the five subtests of the wBCST. This is an important result for the novel test since it indicates good consistency among the wBCST subtests. It could be explained by the uniform visual stimulation material that is used in the subtests (Figure 1). The correlation of the main outcome measure, *S*_*DS*_, and the secondary outcome measures is medium and statistically significant for the speed variation and the lateral acceleration, but only small and not significant for the time spent on brakes, distance to collision, and time to collision. This was also reported by others [35]. However, it is worthwhile to emphasize here that the novel tests show a good correlation of *S*_*wBCST*_ with existing paper-pencil-based cognitive screening tools, such as the MoCA score, the TMT-A, and the TMT-B, but not with the CDT.

The group differences in the wBCST test and its subscores are all significant, which is a prerequisite for using the wBCST to predict DS performance. The AUC of the ROC curve is slightly larger than 0.80, which is generally considered a good test [33,36]. The ROC curve (Figure 5) and the corresponding table with sensitivity, specificity, and efficiency values allows for selecting a cutoff score that leads to the desired test properties. As mentioned in the introduction, the test should have both a high sensitivity and specificity. When considering Table 2, an appropriate cutoff with a high efficiency could be 0.75, which would lead to 83% sensitivity, 70% specificity, and 81% efficiency. These values are far from perfect, but are within what is to be expected for a test measuring multifaceted characteristics of the cognitive ability to drive safely [37-43].

In the present study, the overall test result, *S*_*wBCST*_, is calculated simply as the mean of the subscores, *S*_*1…5*_. It could be that one subtest is more informative than another and, in this case, the subscores should be weighted differently. The sample size of this study is too small to determine the optimal weighting parameters, but this is an interesting question for a future study.

Overall, the novel wBCST was very well accepted by the test population and there were no drop-outs in the wBCST. This is in contrast to the DS with 19 drop-outs (19.1%) due to simulator sickness (18 drop-outs) and technical problems (1 drop-out). Since it is Web-based, the distribution of the test software to different test-sites will be fairly easy and with the central scoring algorithm, data consistency among the different centers can be ensured, which will facilitate validation studies in larger populations.

The online instructions of the test procedure take about 5 minutes. We did not observe difficulties of the test persons to understand the task, except two cognitively impaired test subjects wanted to go through the instructions twice. We concluded that the instructions are clear, but participants should have the option to repeat the instructions.

The steering wheel and the foot pedal are fairly cheap accessories, but to further improve accessibility of the novel test it would be beneficial if the test could also be used with keyboard and mouse. We observed that steering wheel and foot pedal seem to increase the face-validity of the tests, but we would expect that the measured cognitive functions should be independent from the input modality. This could be investigated in a future study.

### Limitations

This pilot study has some limitations that need to be mentioned. One is the large number of drop-outs in the DS that might create a selection bias, since it cannot be excluded that people with poor driving performance might be more prone to simulator sickness. Although the current literature on simulator sickness suggests other contributing factors (eg, age, gender), this cannot be excluded [20]. Another limitation is that there are no published and accepted cutoff values for the DS, which makes the selection of the cutoff for the group differentiation difficult. Compared to the DS evaluation, the administration of the wBCST takes 20 minutes, which is quite long. In future studies, one could investigate whether or not test duration could be shortened. Furthermore, the test-retest and inter-rater reliability should be assessed in a future study.

### Conclusions

In this pilot study, the novel wBCST looks like a promising test to support clinicians in fitness-to-drive assessments of older drivers. The Web-based distribution and the scoring on a central computer will facilitate further evaluation of the novel test setup. In its current form, the program requires local installation on a client computer in the physician’s office. This is currently not considered a disadvantage, but, of course, one could transfer the test program to run within a Web browser, which would not require local installation. The hardware requirements of the wBCST are very minor and include an office-type personal computer with Windows 7 operating system and a simple steering wheel (eg, Driving Force GT, Logitec Inc). Overall, when considering disadvantages of DS (costs, simulator sickness, space requirements), the authors believe that in many clinical environments the wBCST is better suited to support physicians in fitness-to-drive assessments than a DS. That is why we expect that in the near future, Web-based computer tests will become a valuable and usable tool for fitness-to-drive assessment in older drivers.

## Multimedia Appendix 1

Screencast with animated images from the experimental setup (.wmv File, 44 MB).

## Multimedia Appendix 2

Screencast with animated images from the experimental setup (.mp4 File, 92 MB).

## Abbreviations

AUC: area under the curve
CDT: clock drawing test
DS: driving simulator
MoCA: Montreal Cognitive Assessment
ORT: on-the-road testing
ROC: receiver operating characteristic curve
TMT A/B: trail making tests A/B
wBCST: Web-based Bern Cognitive Screening Test
